# Research on system of ultra-flat carrying robot based on improved PSO algorithm

**DOI:** 10.3389/fnbot.2023.1294606

**Published:** 2023-11-28

**Authors:** Jinghao Zhu, Jun Wu, Zhongxiang Chen, Libo Cao, Minghai Yang, Wu Xu

**Affiliations:** ^1^State Key Laboratory of Advanced Design and Manufacturing Technology for Vehicle, Hunan University, Changsha, China; ^2^College of Engineering and Design, Hunan Normal University, Changsha, China; ^3^Hunan Lizhong Technology Limited Company, Changsha, China

**Keywords:** ultra-flat carrying robot, improved PSO algorithm, system identification, critical proportioning method, optimization of PI parameters

## Abstract

Ultra-flat carrying robots (UCR) are used to carry soft targets for functional safety road tests of intelligent driving vehicles and should have superior control performance. For the sake of analyzing and upgrading the motion control performance of the ultra-flat carrying robot, this paper develops the mathematical model of its motion control system on the basis of the test data and the system identification method. Aiming at ameliorating the defects of the standard particle swarm optimization (PSO) algorithm, namely, low accuracy, being susceptible to being caught in a local optimum, and slow convergence when dealing with the parameter identification problems of complex systems, this paper proposes a refined PSO algorithm with inertia weight cosine adjustment and introduction of natural selection principle (IWCNS-PSO), and verifies the superiority of the algorithm by test functions. Based on the IWCNS-PSO algorithm, the identification of transfer functions in the motion control system of the ultra-flat carrying robot was completed. In comparison with the identification results of the standard PSO and linear decreasing inertia weight (LDIW)-PSO algorithms, it indicated that the IWCNS-PSO has the optimal performance, with the number of iterations it takes to reach convergence being only 95 and the fitness value being only 0.117. The interactive simulation model was constructed in MATLAB/Simulink, and the critical proportioning method and the IWCNS-PSO algorithm were employed respectively to complete the tuning and optimization of the Proportional-Integral (PI) controller parameters. The results of simulation indicated that the PI parameters optimized by the IWCNS-PSO algorithm reduce the adjustment time to 7.99 s and the overshoot to 13.41% of the system, and the system is significantly improved with regard to the control performance, which basically meets the performance requirements of speed, stability, and accuracy for the control system. In conclusion, the IWCNS-PSO algorithm presented in this paper represents an efficient system identification method, as well as a system optimization method.

## 1 Introduction

The increasingly mature intelligent driving technology has brought new safety problems, which has resulted in higher requirements for vehicle safety (Stilgoe, 2021). The ultra-flat carrying robot (UCR) is a kind of road test equipment applied to the functional safety of intelligent driving vehicles, which can be equipped with a variety of soft targets to reproduce road hazard scenarios (Steffan et al., 2017; Uchida and Yamazaki, 2018; Bartholomew et al., 2023). It fits well with the testing needs of industry regulations and compensates for the limitations of previous testing methods. The ultra-flat carrying robot has the characteristics of stability, reliability, easy maintenance, wide use, and low radar properties. In detail, first of all, the ultra-flat carrying robot's upper cover plate and body are of an integrated design, which has stronger load-bearing capacity and higher stability; it adopts the modular design of bottom drive + central control + upper computer, which is more stable in control and more convenient in upgrading. Secondly, the ultra-flat carrying robot can carry soft target dummies, bicycles, and electric scooters, and its trajectory can be customized to meet the test requirements in the New Car Assessment Program (NCAP) and other regulations. Last but not least, the ultra-flat carrying robot adopts the structural design of invisible materials combined with an inclined plane for the first time in the world, which greatly reduces the radar attribute value of the product and effectively avoids the false detection of vehicle sensors in the testing process. The ultra-flat carrying robot usually adopts the Proportional-Integral (PI) control algorithm. For the purpose of enabling the ultra-flat carrying robot to be equipped with superior control performance, it is of vital importance to establish an accurate mathematical model of the motion control system of the ultra-flat carrying robot and optimize the parameters for the PI controller. In general, the parameters of Proportional-Integral-Derivative (PID) controllers are largely dependent on manual experience to try and figure out, which results in a considerable amount of work, wastage of time, and difficulty in achieving the best control effect (Chunchen et al., 2017).

The particle swarm optimization (PSO) algorithm is typically employed to address issues such as system parameter identification and optimization. For multi-parameter identification models and high-dimensional complex optimization problems, the standard PSO algorithm tends to be premature and fall into the local optimum (Cheng et al., 2021), which leads to poor identification accuracy and unsatisfactory optimization results. In order to strengthen the performance of PSO algorithms, many scholars at home and abroad have actively explored and applied it to the system parameter identification and optimization of PID parameters. Their studies have been summarized in Table 1 for better readability. Ebrahimi et al. (2019) proposed a flexible PSO algorithm to estimate the parameters of the photovoltaic cell model and verified that the algorithm has high accuracy and robustness through three different solar modules. Wang B. M. et al. (2021) presented a random weight PSO algorithm to identify the dynamics parameters of the robot, and the simulation results showed that the random weight PSO algorithm has high accuracy for the identification of robot dynamics parameters. Liu and Zhang (2022) put forward a loop-iteration PSO algorithm to identify multiple parameters of the electric vehicle wireless charging system, which can achieve high identification accuracy under the condition of less detection. Kang and Sun (2023) combined the PSO with spatial disturbance to form an improved PSO algorithm, which realized the integrated identification of the asynchronous motor and load parameters. Chen et al. (2023) adopted an improved chaotic PSO algorithm integrated with the principle of elite immunity to identify the parameters of the permanent magnet synchronous motor online, and verified the effectiveness of the algorithm through simulation and comparison. Wang Q. L. et al. (2021) used a hybrid PSO algorithm that introduced the concept of hybridization in genetic algorithms to optimize the PID controller parameters of automated guided vehicles, which achieved a good control effect. Xiang and Chen (2022) proposed a modified cloud theory-based PSO algorithm introducing cloud evolution and mutation methods to set the initial PID control parameters and optimize the control rules of the fuzzy PID controller, thereby dramatically suppressing oscillation and overshoot. Lv et al. (2022) designed an immune PSO algorithm by improving the inertia weight, learning factor, and particle learning mode and introducing the artificial immunity idea, and then optimized the PID controller. It was verified that the immune PSO algorithm has a better optimal control effect in the simulation environment.

**Table 1:** Summary of the literature review.

**References**	**Research objects**	**Strategies**	**Dimensions**	**Complicated or not**	**Purposes**
Ebrahimi et al. (2019)	Photovoltaics (PV) solar cells and modules	Elimination + search space change	5 and 7	No	Identification
Wang B. M. et al. (2021)	Selective Compliance Assembly Robot Arm (SCARA) robot	Random weight	4	No	Identification
Liu and Zhang (2022)	Electric vehicle wireless	Loop-iteration	4	Yes	Identification
Kang and Sun (2023)	Asynchronous motor and load	Space disturbance	9	No	Identification
Chen et al. (2023)	Permanent magnet synchronous motor	Chaotic algorithm + elite immunity principle	3	Yes	Identification
Wang Q. L. et al. (2021)	Automated guided vehicle	Hybridization concept	3	Yes	Optimization
Xiang and Chen (2022)	Fuzzy PID controller	Cloud evolution and mutation methods	3	Yes	Optimization
Lv et al. (2022)	PID controller	Learning factor change + particle learning mode change, etc. + artificial immunity idea	3	Yes	Optimization
Zhu et al. (this paper)	Ultra-flat carrying robot	Inertia weight cosine adjustment + natural selection principle	7 and 3	No	Identification and optimization

The above studies have resulted in an enhanced performance of the PSO algorithm for system parameter identification and parameter optimization to varying degrees. Nevertheless, there exist issues such as the insufficient performance of the algorithm under multiple parameters, the added sophistication to the improved algorithm, and the enormous computational effort of the algorithm itself and the long time taken. Furthermore, their research objects are mostly low-order systems, while there are comparatively few domestic studies conducted regarding the development of intelligent driving test equipment such as ultra-flat carrying robots at present, and the control performance of ultra-flat carrying robots still need to be further improved.

**Figure 1:**
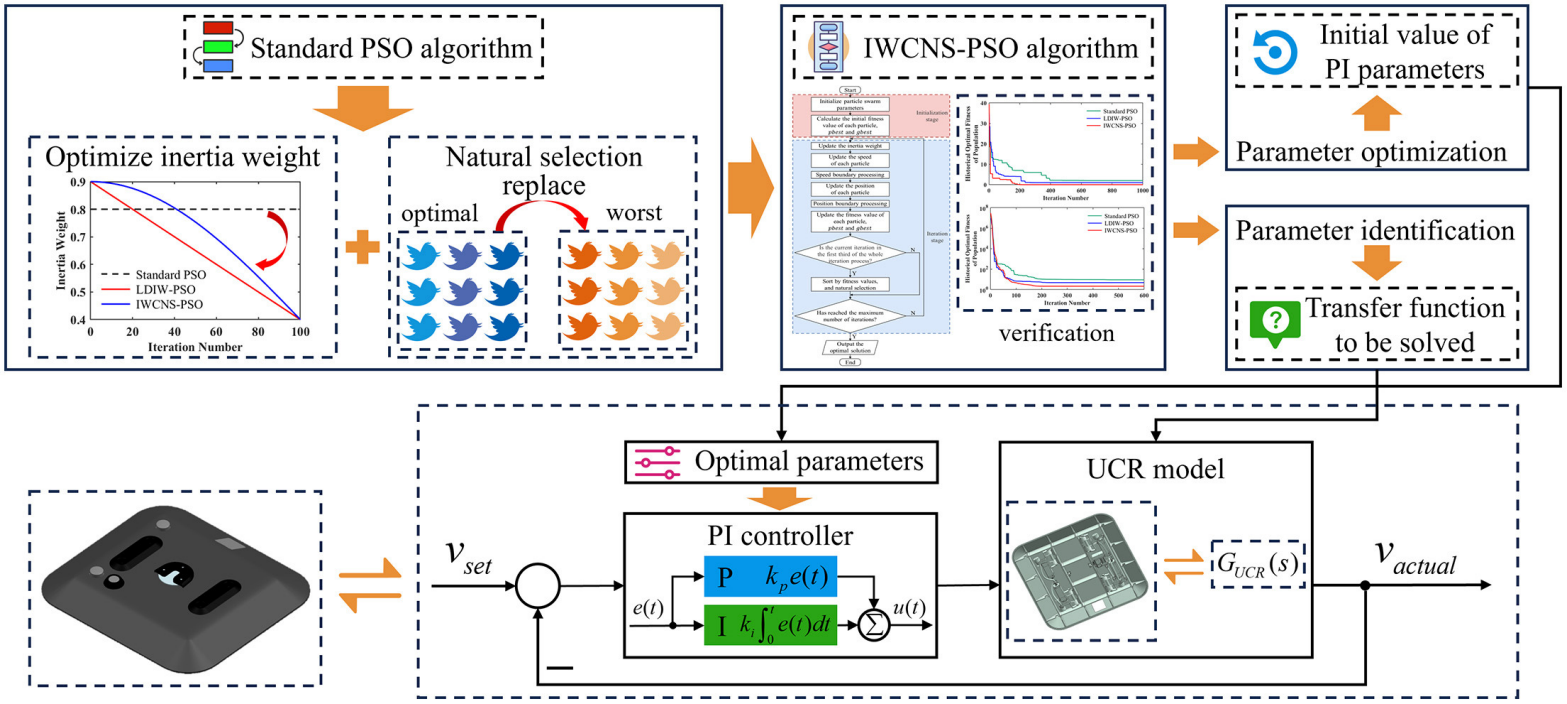
Control method of the ultra-flat carrying robot.

In light of the above issues, taking the ultra-flat carrying robot as the research subject, this paper proposes an improved PSO algorithm called the IWCNS-PSO and applies it to the system parameter identification and optimization of PI parameters, as shown in Figure 1. The lower left corner of Figure 1 refers to the entity UCR, and the lower right corner (the box named “UCR model”) of Figure 1 shows roughly the internal hardware structure of the ultra-flat carrying robot, which contains steering wheels (front wheels), driving wheels (rear wheels), motors, electric push cylinders, torsion springs, and so on. In this paper, the mathematical model of the motion control system of the ultra-flat carrying robot is firstly established and determines the parameters to be identified. Afterwards, for the purpose of accurately identifying the unknown parameters in the model, the IWCNS-PSO algorithm is proposed in this paper, while verifying the performance of the IWCNS-PSO via test functions and comparison with the standard PSO, LDIW-PSO, Ant Colony Optimization (ACO), and Simulated Annealing (SA), thereby highlighting the superiority of the IWCNS-PSO algorithm. Thereafter, on the basis of the IWCNS-PSO algorithm, with the sum of squares due to error (*SSE*) being selected as the fitness function, this paper completes the transfer function identification for the motion control system of the ultra-flat carrying robot, in addition to comparing the identification results of the other two PSO algorithms. Finally, by employing a MATLAB/Simulink interactive simulation, this paper adopts the critical proportioning method and the IWCNS-PSO algorithm respectively with *ITAE* as the fitness function to tune and optimize the PI controller parameters, which improves the speed, accuracy, and stable control performance of the system. The IWCNS-PSO algorithm introduced in this paper represents an efficient system identification and system optimization method with a certain degree of value for engineering applications.

The principal contributions of this paper are outlined as follows:

(1) The fourth-order mathematical model of the motion control system in the ultra-flat carrying robot, which is consistent with the features of the test data, is established by means of the system identification method, and the seven unknown parameters to be identified are determined.
(2) With the inspiration of the standard PSO and LDIW-PSO, this paper proposes the IWCNS-PSO with inertia weight cosine adjustment and introduction of natural selection principle. With the Rastrigin function and Rosenbrock function, the performance of this algorithm is analyzed in comparison with the other two PSO algorithms and the other two heuristic algorithms. Both the speed and accuracy of optimization of the PSO algorithm have been remarkably improved.
(3) The transfer function of the motion control system in the ultra-flat carrying robot is accurately identified using the IWCNS-PSO algorithm. An interactive simulation model of optimizing PI parameters with the IWCNS-PSO algorithm is built in MATLAB/Simulink. The optimized PI parameters reduce the adjustment time to 7.99 s and the overshoot to 13.41% of the system, which significantly enhances the control performance of the system.
(4) This paper compares and analyzes the tuning and optimization results of the PI controller parameters obtained by the critical proportioning method and the IWCNS-PSO algorithm, and proposes to combine the two for applications in engineering practice. To a certain extent, this paper provides theoretical guidance for the development and debugging of similar electromechanical products such as ultra-flat carrying robots.

## 2 Proposed methodology

### 2.1 System identification-based modeling of ultra-flat carrying robots

**Figure 2:**
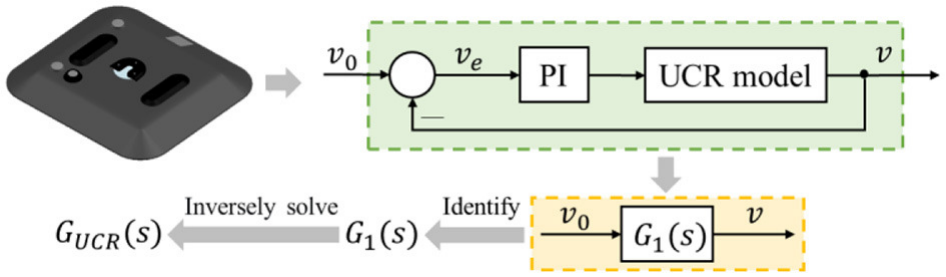
Research idea.

With the aim of quantitatively analyzing and optimizing a control system, it is imperative to first develop its mathematical model. The motion control chassis of the ultra-flat carrying robot consists of the control module, drive subsystem, brake motor, etc., which is a comparatively complex system with greater difficulty in modeling by theoretical analysis methods. System identification involves no in-depth insight into the internal mechanisms of the system, which exploits the information available from the input and output data to build a mathematical model of the system for use in system prediction and design, among other things (Ljung, 1987; De Persis and Tesi, 2019). By adopting the system identification method, this paper establishes the mathematical model of the motion control system in the ultra-flat carrying robot and obtain its transfer function, with the research idea illustrated in Figure 2.

The motion control chassis of the ultra-flat carrying robot is a speed closed-loop control system, which can be equated to the form shown in the lower box in Figure 2, whose equivalent transfer function *G*_1_(*s*) is termed as the closed-loop transfer function. Meanwhile, this system represents a unit negative feedback system, thereby, *G*_1_(*s*) can be expressed as:


(1) $$\begin{array}{l} G_{1}\left(S\right)\;=\;\frac{G_{PI\;+\;UCR}\;\left(S\right)}{1\;+\;G_{PI\;+\;UCR}\;\left(S\right)} \end{array}$$

**Figure 3:**
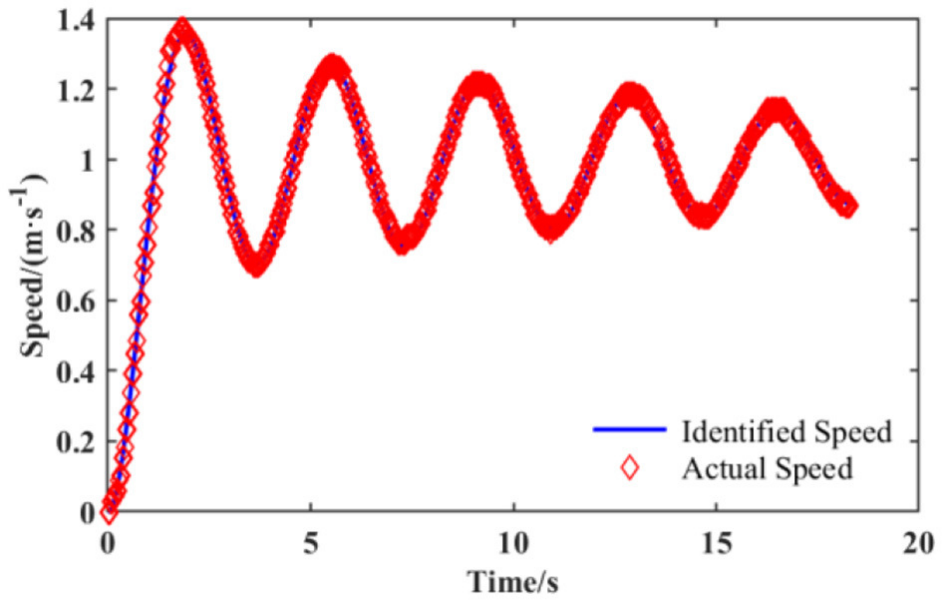
Satisfactory fitting effect of the identification data and the actual data.

The time-domain performance indexes of the control system are determined in accordance with the output response of the system subjected to a unit step signal with zero initial condition (Zheng et al., 2017). Therefore, this paper selects the unit step speed signal as the input signal for the ultra-flat carrying robot during the test, while its actual response speed of the output is recorded with the inertial navigation (Collin et al., 2019). For the sake of ensuring the fitting degree between the output series of the established mathematical model and the actual data, it is crucial to choose the appropriate form of the transfer function. The satisfactory fitting effect should be as shown in Figure 3. In consideration of the fact that the unit step response in a second-order system normally does not match with Figure 3, it is considered to increase the order of the system to enhance the fitting effect. By combining experience and previous attempts, the number of poles of the transfer function to be identified are chosen to be four and the number of zeros to be two. The form of it is as follows:


(2) $$\begin{array}{l} G_{1}\left(s\right)=\frac{\lambda s^{2}+\mu s+\varphi }{s^{4}+\alpha s^{3}+\beta s^{2}+\gamma s\;+\delta } \end{array}$$

where α, β, γ, δ, λ, μ, and φ are the parameters to be identified.

First and foremost, the PSO algorithm and test data are employed to identify the closed-loop transfer function *G*_1_(*s*) of the motion control system in the ultra-flat carrying robot, followed by deriving the equivalent transfer function *G*_*PI*+*UCR*_(*s*) of the PI controller and the UCR model in series according to the inverse of Equation 1. Under the condition that the transfer function of the PI controller is known, the transfer function *G*_*UCR*_(*s*) of the UCR model is available to be obtained and applied to the subsequent tuning and optimization of the PI controller parameters.

### 2.2 Standard PSO algorithm

Particle swarm optimization (PSO) refers to a swarm intelligence algorithm originally introduced by Eberhart and Kennedy (1995) and Kennedy and Eberhart (1995), which was motivated by some of the social behaviors of animals, such as foraging in flocks of birds. As a stochastic search method, it features the advantages of good robustness, easy implementation, low parameter settings, and small memory space occupation (Jain et al., 2022), while being widely applied in system optimization, neural network training, pattern recognition, and other fields (Singh et al., 2022).

The PSO algorithm firstly initializes a set of particles without mass randomly in the search space, while each particle stands for a feasible solution in space, which gets the corresponding fitness value by being substituted into the objective function. In the search for the optimal solution, the particles continuously renew their speeds and positions with reference to the optimal position they have reached and follow the particle with the optimal position in the population, thereby seeking the optimal solution in the solution space. It is assumed that the dimension of the solution space is *d* while the size of the population is *n*, then the speed and position for the *i*-th particle can be written as:


(3) $$\begin{array}{l} v_{i}=\left(v_{i1},v_{i2},...,v_{id}\right),i\;=\;1,2,...,d \end{array}$$

(4) $$\begin{array}{l} x_{i}=\left(x_{i1},x_{i2},...,x_{id}\right),i\;=\;1,2,...,d \end{array}$$

This algorithm keeps two optimal positions all along: one is the optimal position of the *i*-th particle passed so far and the other is the optimal position for all the particles in the whole population, which are denoted by *pbest*_*i*_ and *gbest*, respectively. In other words, *pbest* and *gbest* stand for the optimal location historically and globally in the *k*-th iteration. Furthermore, *pbest*_*i*_ of the *i*-th particle corresponds to its historical best fitness value, while all particles share the position (*gbest*) of global best fitness value. Upon locating these two optimal values, the particle updates its speed and position in each iteration in line with the following equations:


(5) $$\begin{array}{l} v_{i}^{k+1}=\omega v_{i}^{k}+c_{1}r_{1}\left(pbest_{i}^{k}-x_{i}^{k}\right)+c_{2}r_{2}\left(gbest-x_{i}^{k}\right) \end{array}$$

(6) $$\begin{array}{l} x_{i}^{k+1}=x_{i}^{k}+v_{i}^{k+1} \end{array}$$

where *k* refers to the number of current iteration; ω denotes the inertia weight; and *c*_2_ denote the cognitive learning factor and social learning factor of the particle, respectively, which normally take values between $0\tilde{\;}2$ (Xia and Li, 2020), signifying the magnitude of the influence exerted by the experience of the particle itself and the population on the position movement of this particle; and *r*_1_ and *r*_2_ represent two numbers between [0, 1] that are generated randomly. It is precisely through the synergistic cooperation and information sharing among the particles that they decide the next movement (Shu et al., 2021).

### 2.3 IWCNS-PSO algorithm

In the PSO algorithm, the larger the inertia weight ω is, the wider the particle's search range and the more robust the global optimization ability will be (Bhattacharya et al., 2019) while the more feeble the local optimization capability will become; the smaller the ω is, the more feeble the particle's global optimization ability will be while the more robust the local optimization ability will become. In the standard PSO algorithm, ω is a fixed value. Therefore, the performance of the PSO algorithm can be optimized by means of adjusting the inertia weight ω.

**Figure 4:**
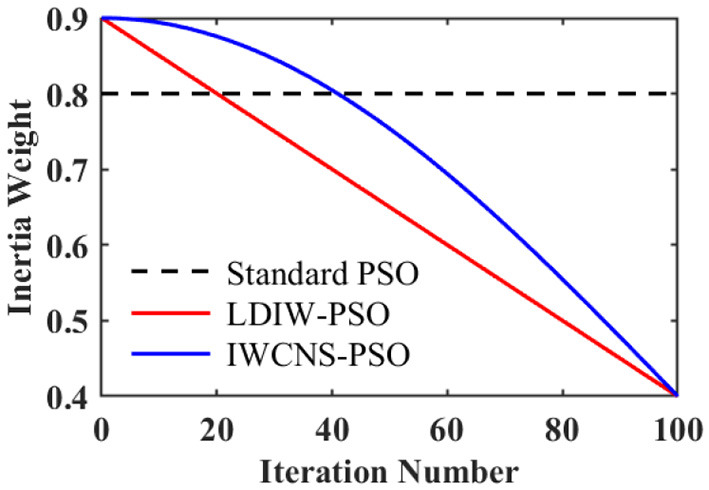
Inertia weight changes of the standard PSO, LDIW-PSO, and IWCNS-PSO.

Shi and Eberhart (1998) put forward the strategy of linear decreasing inertia weight (LDIW), as shown in Figure 4, which can realize the process of changing from a stronger global search at the initial stage to a stronger local search at the late stage, and can achieve better solution accuracy to a certain extent compared with ω as a fixed value. However, in this strategy, the change rate of the inertia weight is a fixed value, so that the numbers of iterations of the particle swarm in the more robust global search and the more robust local search are essentially identical, thereby the global optimal solution is likely not to be found in the global search at the early stage of the iteration, and is likely not to better approach the global optimal solution in the local search at the late stage of the iteration.

**Figure 5:**
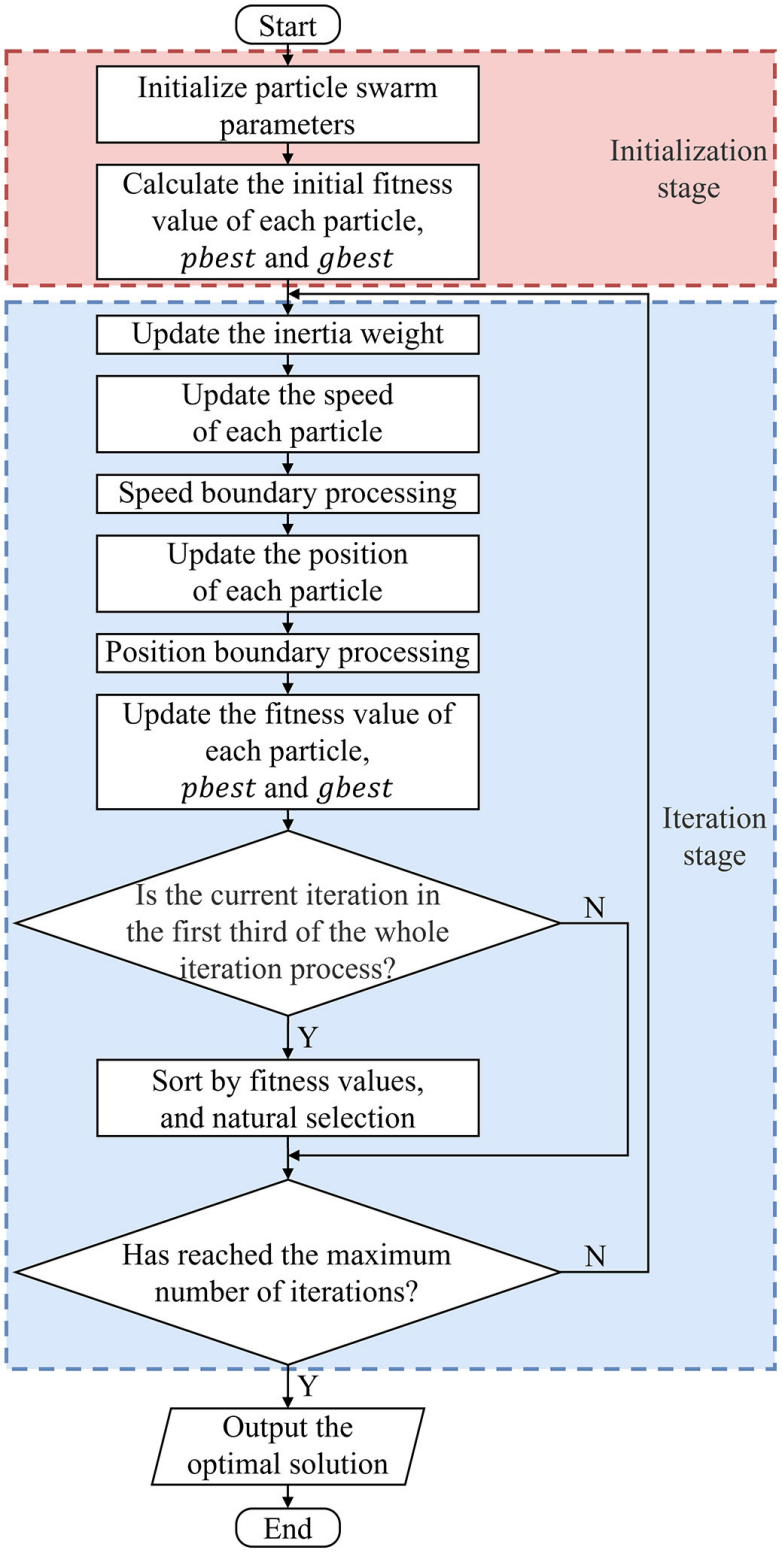
IWCNS-PSO algorithm flow chart.

Based on this, this paper further optimizes the inertia weight by adjusting the change rate of the inertia weight to a dynamic change and proposes an improved PSO algorithm (IWCNS-PSO) with inertia weight cosine adjustment and the introduction of the principle of natural selection. The improved inertia weight is expressed as:


(7) $$\begin{array}{l} \omega (k)=\omega_{\min}\left(1-\cos\left(\frac{\pi k}{2k_{\max}}\right)\right)+\omega_{\max}\cos\left(\frac{\pi k}{2k_{\max}}\right) \end{array}$$

where and ω_min_ = 0.4. The change of the inertia weight is shown in Figure 4. During the initial stage of the search, ω is larger and its change rate is slower, which is beneficial for the particle swarm to conduct a long-term global search and jump out of the local optimum. Consequently, the likelihood of finding the global optimal solution is substantially enhanced. In the late stage of the search, ω is smaller and its change rate is faster, which strengthens the ability of particles to approach the global optimal solution and further improves the solution accuracy of the algorithm. In addition, in the first third period of the whole iteration process, ω changes slowly. In order to further accelerate the convergence, a principle similar to the natural selection in genetic algorithms is introduced: after each iteration is completed, all the particles are sorted by fitness values, and the speed and position of the same number of the worst particles are substituted with those of the optimal 25% of particles in the population, keeping *pbest* and *gbest* unchanged. In this way, the proportion of optimal particles in the population can be increased, and the performance of the particles in each iteration is better. As a result, the convergence speed is accelerated. The integrated flow of the IWCNS-PSO algorithm is illustrated in Figure 5.

The detailed implementation steps of the IWCNS-PSO algorithm are described as follows:

Step 1: Set the initial parameters of the IWCNS-PSO algorithm, such as *c*_1_, *c*_2_, ω_min_, ω_max_, etc. Randomly initialize the speed and position of each particle in the population;
Step 2: Calculate the initial fitness value of each particle according to the objective function, store the current position of each particle in respective *pbest*, and store the position of the particle with the optimal fitness value in *gbest*;
Step 3: Update the inertia weight in accordance with Equation 7;
Step 4: Update the speed of each particle in accordance with Equation 5;
Step 5: Judge whether the *s*peed of the particle is beyond the limited range. If it exceeds this, the speed is modified to the corresponding boundary value; otherwise, it remains unchanged;
Step 6: Update the position of each particle in accordance with Equation 6;
Step 7: Judge whether the position of the particle is beyond the limited range. If it exceeds this, the position is modified to the corresponding boundary value; otherwise, it remains unchanged;
Step 8: Update the fitness value of each particle. If the particle's current fitness value is better than its historical optimal fitness value, update its historical optimal fitness value to the current fitness value, and update the particle's current position to *pbest*, otherwise it remains unchanged. Update the position of the particle with the optimal fitness value in the whole population to *gbest*;
Step 9: If the current iteration number is in the first third of the maximum number of iterations, sort all the particles in line with the current fitness values and replace the speed and position of the identical number for the worst particles with those of the optimal 25% of particles, keeping *pbest* and *gbest* unchanged; otherwise, skip Step 9;
Step 10: If the maximal number with iterations has been attained, output the optimal solution *gbest*, and the algorithm ends; otherwise, return to Step 3.

## 3 Comparison and verification of algorithm performance

The IWCNS-PSO algorithm is refined by inertia weight cosine adjustment strategy, while the quality of the particles in the algorithm is optimized by the principle of natural selection. Through the fitness value and iteration time (Xia and Li, 2020), this paper assesses the merits and demerits of the algorithms, so as to embody the effectiveness of the proposed IWCNS- PSO algorithm in complex optimization problems, as well as the fact that the IWCNS-PSO algorithm features the best effect, which is relative to the other two PSO algorithms and the other two heuristic algorithms.

As an essential indicator of the performance of intelligent optimization algorithms, the convergence curve can intuitively reflect whether the algorithm falls into a local optimal solution, as well as the time and the number of times it falls into a local optimal solution. To verify the superiority of the IWCNS-PSO algorithm, the Rastrigin function and Rosenbrock function are selected typically for testing (Fischer et al., 2023), in which the convergence curves of the IWCNS-PSO algorithm are in comparison with the convergence curves of the standard PSO algorithm, LDIW-PSO algorithm, simulated annealing (SA) algorithm, and ant colony optimization (ACO) algorithm. The specific equation of the Rastrigin function is:


(8) $$\begin{array}{l} f\left(x\right)=\overset{n}{\underset{i=1}{\sum }}\left({x_{i}}^{2}-10\cos\left(2\pi x_{i}\right)+10\right),\;x\in \left[-5.12,5.12\right] \end{array}$$

**Figure 6:**
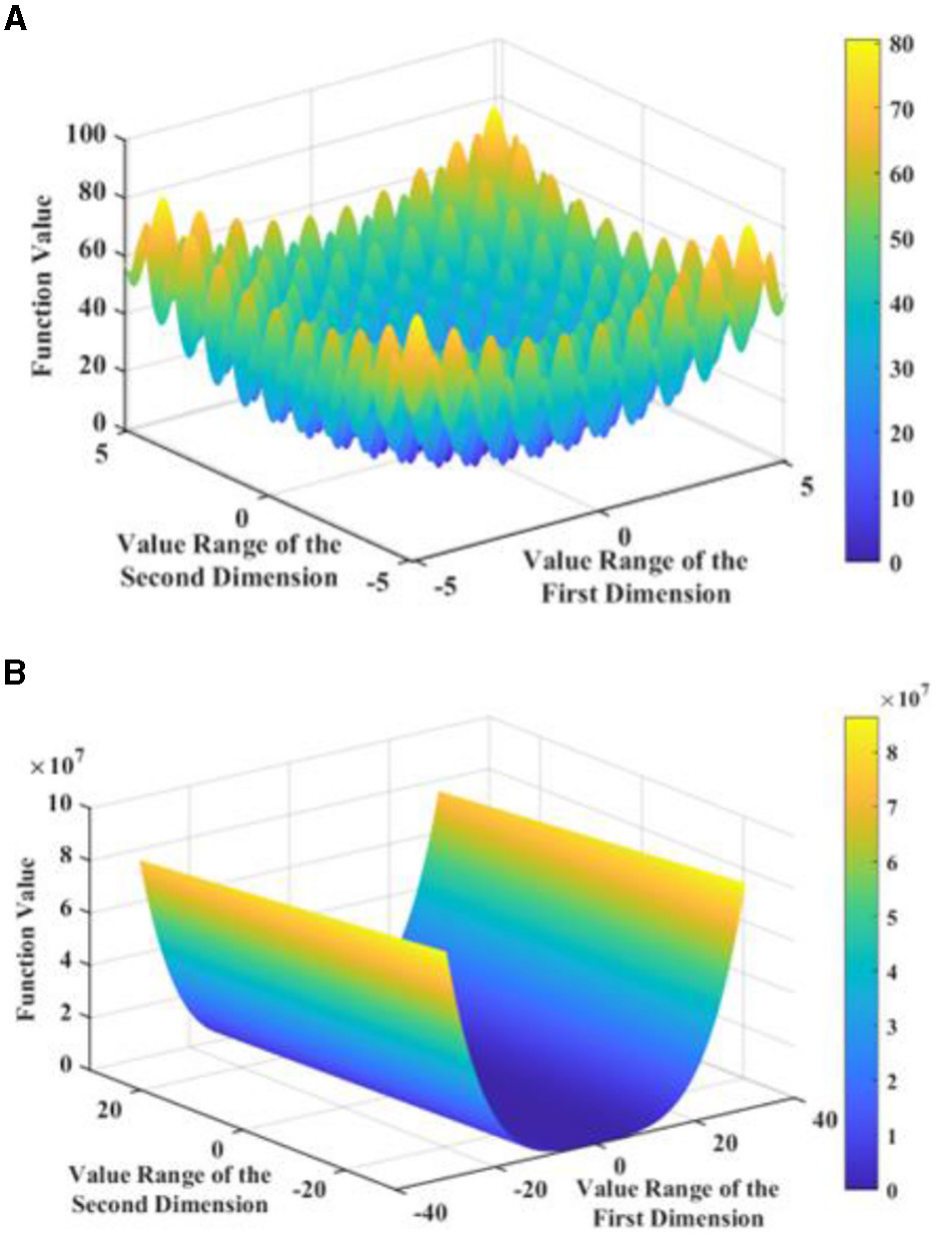
Images of the two functions. **(A)** Two-dimensional Rastrigin functional. **(B)** Two-dimensional Rosenbrock functional.

The specific equation of the Rosenbrock function is:


(9) $$\begin{array}{l} f\left(x\right)=\overset{n-1}{\underset{i=1}{\sum }}\left[100{\left(x_{i+1}-{x_{i}}^{2}\right)}^{2}+{\left(x_{i}-1\right)}^{2}\right]\;,x\in \left[-30,30\right] \end{array}$$

where *n* is the number of variables, namely, the dimensionality of the solution space. The Rastrigin function is a non-linear multimodal function with multiple local extrema and reaches its global minimum of 0 when all variables are 0. The Rosenbrock function is a non-convex pathological function that is difficult to converge to the global minimum and reaches its global minimum of 0 when all variables are 1. The images of the two-dimensional Rastrigin function and Rosenbrock function are shown in Figure 6.

**Figure 7:**
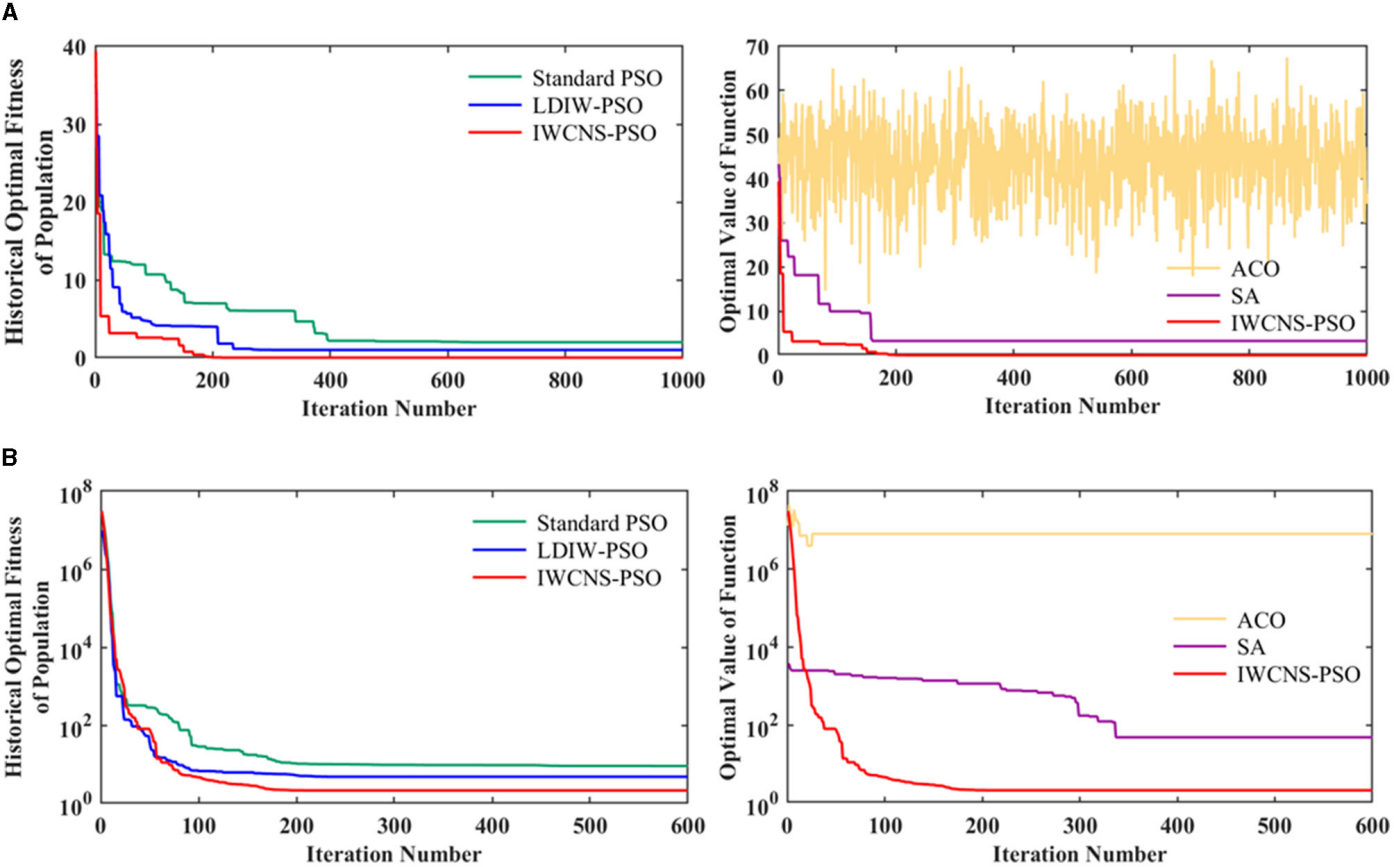
Fitness convergence curves of the standard PSO, LDIW-PSO, IWCNS-PSO, ACO, and SA. **(A)** Rastrigin. **(B)** Rosenbrock.

The color bar and its gradient in Figure 6 intuitively show the complexity of the test function and the distribution and range of the function values, from which it can be easily seen that the global minimum value of the function is 0. The 5-dimensional Rastrigin function and the 10-dimensional Rosenbrock function were chosen to test the performance of the standard PSO, LDIW-PSO, IWCNS-PSO, SA, and ACO in processing high-dimensional complex optimization problems. The fitness functions or objective functions are naturally the Rastrigin function and Rosenbrock function, respectively, the value range *x*_lim_ of solution is limited to [−5.12, 5.12] and [−30, 30], and the dimension of solution space *d* is set to 5 and 10. For the PSO, the initial parameters of the particle swarm are set as follows: the population size *n* is 50, the maximum number of iterations *k*_max_ is 1,000 and 600, the speed limit *v*_lim_ is [−2, 2], the fixed initial inertia weight is ω = 0.8 in the standard PSO algorithm and the initial inertia weight is ω_max_ = 0.9 and the ending inertia weight is ω_min_ = 0.4 in the LDIW-PSO and IWCNS-PSO algorithms, the cognitive learning factor *c*_1_ and the social learning factor *c*_2_ of the particles are both 2, the population's initial speed is *v* = *rand*(*n, d*), and the initial position is *x* = *x*_lim_(1)+[*x*_lim_(2)−*x*_lim_(1)]·*rand*(*n, d*). For the SA, the initial temperature *T* is set to 100, the final temperature is set to 10^−8^, the cooling rate is set to 0.99, and the Markov chain length is set to 300. For the ACO, the number of ants is set to 50, the maximum number of iterations is set to 1,000 and 600, the pheromone importance factor α is set to 2, the heuristic factor β is set to 4, the pheromone evaporation coefficient ρ is set to 0.2, and the pheromone increment *Q* is set to 10. The fitness convergence curves of the five algorithms are shown in Figure 7.

As can be observed from Figure 7, the ACO shows the worst results, followed by the SA, which means that the ACO and SA may be inefficient in processing high-dimensional complex optimization issues compared with PSO algorithms. In the three PSO algorithms, the standard PSO shows the slowest convergence speed, and the convergence result is very different from the actual minimum of the function, which indicates that the standard PSO is caught in the local optimum. As a consequence, the standard PSO is highly susceptible to fall into the local optimum with low accuracy and slow convergence of fitness when applied to multi-parameter problems. In comparison with the standard PSO, the performance of the LDIW-PSO has been improved to a certain extent with a faster convergence speed and a convergence result closer to the actual minimum of the function. However, it is still caught in the local optimum. Apparently, the IWCNS-PSO not only converges the fastest, but also converges the result closest to the actual minimum of the function or is even consistent with it, achieving the highest convergence accuracy.

In short, the test results indicate that the improvements proposed in this paper to the standard PSO algorithm are effective. In contrast to the standard PSO, LDIW-PSO, ACO, and SA (the other two heuristic algorithms), the IWCNS-PSO has been considerably improved for its optimization capability. With respect to most optimization issues, the IWCNS-PSO algorithm outperforms the other four algorithms. The IWCNS-PSO algorithm has high optimization efficiency and prominent advantages.

## 4 Results and analysis of transfer function identification of ultra-flat carrying robots based on the IWCNS-PSO algorithm

The IWCNS-PSO algorithm is applied to identify the unknown parameters in the transfer function (Equation 2) of the motion control system of the ultra-flat carrying robot, and the identification results are compared with those of the standard PSO algorithm and LDIW-PSO algorithm. Set *n* = 100, *d* = 7, *k*_max_ = 300. Additionally, the ranges of the seven unknown parameters to be identified equivalent to *x*_lim_ in Section 3 are all set to [0, 8], and the rest of the parameters are the same as those in Section 3. The fitness function is chosen as the sum of squares due to error (*SSE*):


(10) $$\begin{array}{l} SSE_{i}^{k}=\overset{T}{\underset{t=0}{\sum }}\left\{{\left(y\left(t\right)-y_{i}^{k}\left(t\right)\right)}^{2}\right\} \end{array}$$

where *t* is a series of discrete time sequence from 0 to *T*; *y*(*t*) is the actual output sequence of the system; and $y_{i}^{k}$ is the system output sequence corresponding to the transfer function obtained for the *i*-th particle after the *k*-th iteration. The PSO algorithms are employed to identify the unknown parameters of the transfer function, namely, to obtain the position of the particle corresponding to the historical optimal fitness of the population that is expressed as *gbest* before when the maximum number of iterations is reached, which satisfies the minimum sum of squares due to error. The identification results of the standard PSO, LDIW-PSO, and IWCNS-PSO are presented in Table 2.

**Table 2:** Identification results of the standard PSO, LDIW-PSO, and IWCNS-PSO.

**Algorithms**	**α**	**β**	**γ**	**δ**	**λ**	**μ**	**φ**	**SSE**	**Number of times**
Standard PSO	2.730	6.038	8	8	2.845	3.519	8	0.197	229
LDIW-PSO	1.943	5.856	5.630	8	2.932	2.072	7.981	0.145	158
IWCNS-PSO	2.185	5.883	6.359	7.923	2.881	2.504	7.916	0.117	95

**Figure 8:**
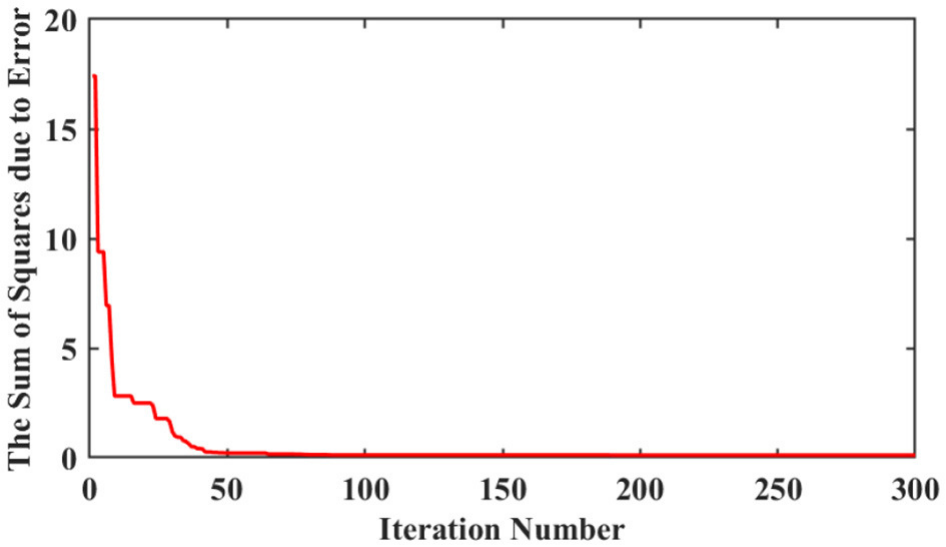
Fitness convergence curve of the IWCNS-PSO.

As can be observed from Table 2, the IWCNS-PSO algorithm corresponds to the smallest sum of squares due to error, which suggests that it features the highest identification accuracy and the best fitting effect. Moreover, the IWCNS-PSO also requires the least number of iterations to reach the convergence of the population's historical optimal fitness, whose fitness convergence curve is shown in Figure 8, which embodies again the superiority of the IWCNS-PSO algorithm compared with the standard PSO algorithm and LDIW-PSO algorithm. The identified transfer function *G*_1_(*s*) is given by:


(11) $$\begin{array}{l} G_{1}\left(s\right)=\frac{2.881s^{2}+2.504s+7.916}{s^{4}+2.185s^{3}+5.883s^{2}+6.359s+7.923} \end{array}$$

In accordance with the coefficients of the characteristic equation, the Routh table of this fourth-order system is established as Table 3.

**Table 3:** Routh table of the fourth-order system.

*s* ^4^	1.000	5.883	7.923
*s* ^3^	2.185	6.359	0
*s* ^2^	2.973	7.923	
*s* ^1^	0.535	0	
*s* ^0^	7.923		

According to the Routh criterion (Li et al., 2019), all the elements in the first column of the Routh table are >0, which means that this fourth-order system is stable. The transfer function *G*_*UCR*_(*s*) of the UCR model can be further derived from Equation 1, which will be applied to the tuning and optimization of the PI controller parameters hereafter.

## 5 PI parameter optimization based on the IWCNS-PSO algorithm

### 5.1 Principle of optimizing PI parameters using the IWCNS-PSO algorithm

PID control adjusts the controlled object through the linear combination of the proportional (P), integral (I), and differential (D) of the error to constitute the control quantity. It features the advantages of a simple structure and strong robustness, as well as convenient adjustment (Borase et al., 2021). The ultra-flat carrying robot adopts PI control, and its working principle is:


(12) $$\begin{array}{l} u(t)=k_{p}e(t)+k_{i}\int_{0}^{t}e(t)dt \end{array}$$

where *u*(*t*) refers to the output of the controller; *e*(*t*) is the error signal; and *k*_*p*_ and *k*_*i*_ denote the proportional coefficient and integral coefficient, respectively. Laplace transform is carried out on Equation 12 and then the transfer function of the PI controller is obtained as follows:


(13) $$\begin{array}{l} G\left(s\right)=\frac{U\left(s\right)}{E\left(s\right)}=k_{p}+\frac{k_{i}}{s} \end{array}$$

**Figure 9:**
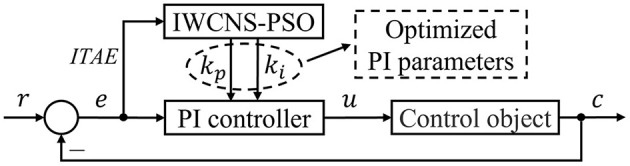
Structure of the PI control system based on the IWCNS-PSO algorithm.

By adjusting *k*_*p*_ and *k*_*i*_, the control performance of the system can meet the requirements as much as possible, so it is crucial to tune and optimize the two parameters. The manual tuning method is based on engineering experience, and it is often difficult to find the global optimal solution when the parameter space is relatively large. The IWCNS-PSO algorithm is a highly efficient search algorithm because of its conciseness, clearness, high optimization accuracy, and fast convergence speed. The structure of the PI control system on the basis of the IWCNS-PSO algorithm is shown in Figure 9.

ITAE is a good performance index (Martins, 2005) that integrates the rapidity, stability, and accuracy of a control system. Many pieces of the literature regard *ITAE* as one of the best performance indexes of single-input single-output control systems and adaptive control systems (Shuaib and Ahmed, 2014). Therefore, it is adopted as the objective function, that is, the fitness function is:


(14) $$\begin{array}{l} ITAE=\int_{0}^{\infty }t|e(t)|dt \end{array}$$

**Figure 10:**
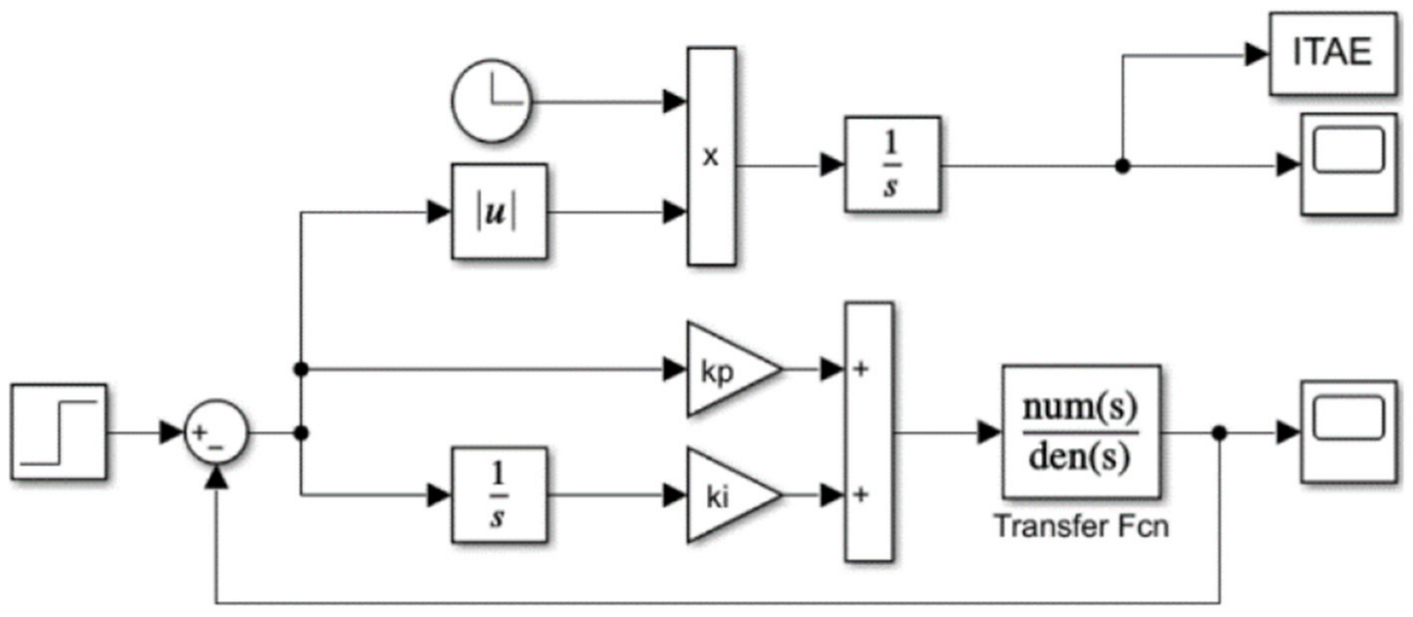
Simulink simulation model.

Utilizing the IWCNS-PSO algorithm to optimize PI parameters is equal to seeking *k*_*p*_ and *k*_*i*_ that minimize *ITAE* (Rao and Santosh, 2020). The simulation model was built in Simulink with reference to Figure 9, as shown in Figure 10, where the input is the unit step signal, the output is the unit step response of the system and the value of *ITAE*, and the Transfer Fcn module corresponds to the transfer function *G*_*UCR*_(*s*) of the UCR model.

### 5.2 Simulation process and results analysis

**Figure 11:**
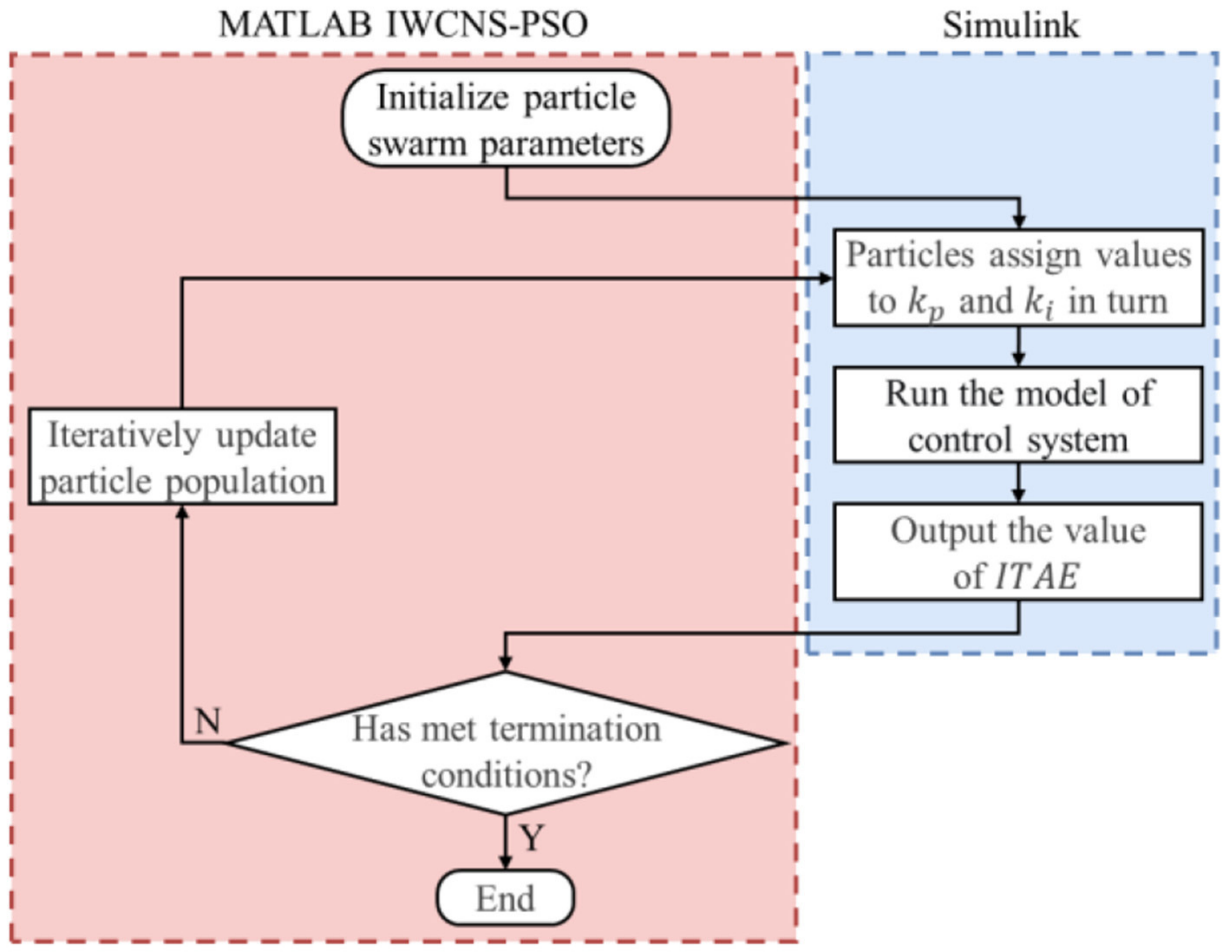
Process of optimizing PI parameters using the IWCNS-PSO algorithm.

This paper performs an interactive simulation by utilizing MATLAB/Simulink version R2021b. The process of optimizing the PI controller parameters based on the IWCNS-PSO algorithm is demonstrated in Figure 11, where the left part (red box) represents the IWCNS-PSO algorithm program (.m file) written in MATLAB and the right part (blue box) represents the simulation model (.slx file) built in Simulink. First and foremost, the particle swarm parameters are initialized in MATLAB, with (*k*_*p*_, *k*_*i*_) representing the spatial position of the population particles, which are assigned to the parameters of the PI controller. Afterwards, the Simulink model is run to obtain the *ITAE* value and input it into the MATLAB workspace. After that, the particle population is updated in accordance with the iterative equations, and and *k*_*i*_ of the new generation of the particles are reassigned to the parameters of the PI controller, while the subsequent process is carried out again, in which the cycle is repeated until the termination condition is satisfied. Eventually, the global optimal solution is obtained, namely, the optimal parameters of the PI controller.

**Figure 12:**
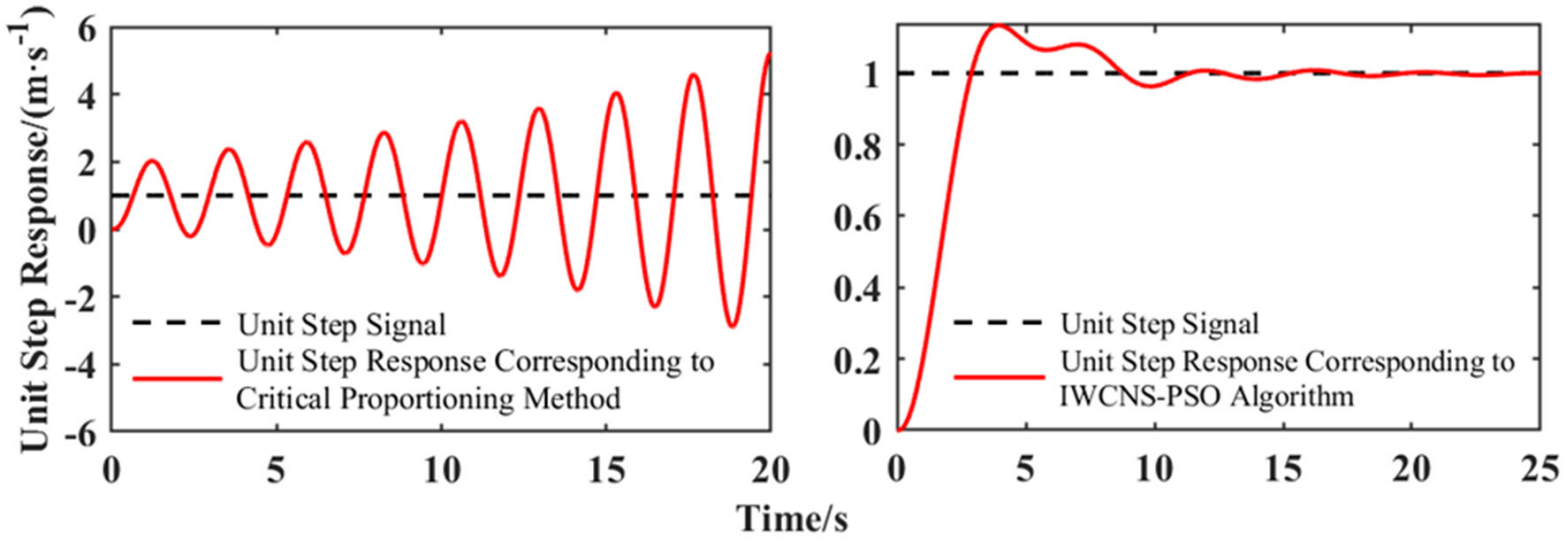
Unit step response.

The critical proportioning method (Shi et al., 2016) and the IWCNS-PSO algorithm are respectively applied to tune and optimize the PI controller parameters. The parameters *n* = 100, *d* = 2, and *k*_max_ = 100 are set, and the ranges of and *k*_*i*_ are set to [0, 500] and [0, 400], respectively, with reference to the results of the critical proportioning method and the influence of *k*_*p*_and *k*_*i*_ on the dynamic performance, while the rest of the parameter settings are the same as those in Section 3. The PI parameters and their corresponding system performance indexes obtained by the critical proportioning method and the IWCNS-PSO algorithm, respectively, are presented in Table 4, while the unit step response of the system is shown in Figure 12.

**Table 4:** Tuning and optimization results of PI controller parameters.

**Parameters and performance indexes**	**Critical proportioning method**	**IWCNS-PSO algorithm**
*k*_*p*_	586.045	40.308
*k*_*i*_	449.508	35.062
Rise time/s	0.65	2.89
Peak time/s	1.22	3.95
Settling time/s	∞	7.99
Overshoot/%	∞	13.41
Steady-state error/%	∞	0
*ITAE* value	∞	5.784

As evidenced by Table 4 and Figure 12, the unit step response that correlates with the PI parameters tuned by the critical proportioning method is oscillatory, divergent, and unrealistic, which fails to meet the needs of the test work. This indicates that the critical proportioning method, which relies on engineering experience, is not suitable for all cases. Meanwhile, the tuning results of the critical proportioning method are likely to make the control performance of the system even more unsatisfactory, especially when dealing with high-order complex systems. By employing the IWCNS-PSO algorithm and referring to the adjustment results of the critical proportioning method, the PI parameters are optimized, which leads to a significant improvement in the control performance, thereby essentially meeting the performance requirements for a speedy, stable, and accurate control system.

As a consequence, in engineering practice, the parameter debugging of the PI/PID controller cannot completely rely on the method of engineering experience. On the basis of the results of the manual tuning method, the IWCNS-PSO algorithm suggested in this paper can further optimize parameters of PI/PID and shrink the parameter debugging ranges. With this as a guideline, the system can then be manually fine-tuned in combination with the actual operation on site, so as to enable the control performance of the system to reach the best and substantially save the debugging time.

## 6 Conclusions

This paper firstly established the fourth-order mathematical model of the motion control system of the ultra-flat carrying robot using the modeling method of system identification, which conforms to the features of the test data. Next, this paper proposed a refined particle swarm optimization algorithm named the IWCNS-PSO that is combined with inertia weight cosine adjustment and the introduction of the natural selection principle with the aim of reducing the defects of the standard PSO algorithm, including low accuracy, being easy to lapse into a localized optimum, and slow convergence when dealing with the parameter identification problems of complex systems. The effectiveness of the improvements of the algorithm and the superiority of the IWCNS-PSO algorithm were verified by comparison and typical test functions. Afterwards, the IWCNS-PSO algorithm was employed to identify the transfer function of the motion control system in the ultra-flat carrying robot, which once again embodied the superiority of the IWCNS-PSO algorithm in comparison with the standard PSO algorithm and the LDIW-PSO algorithm. Thereafter, the critical proportioning method and the IWCNS-PSO algorithm were successively employed in the tuning and optimization of the PI controller parameters. In the interactive simulation environment of MATLAB/Simulink, compared with the tuning effect that is oscillatory and divergent caused by the critical proportioning method, the PI parameters optimized by the IWCNS-PSO algorithm reduced the adjustment time to 7.99 s and the overshoot to 13.41% of the system under the action of the unit step signal. The control performance of the system is obviously improved, and the system can achieve the expected effect quickly, stably, and accurately in consequence.

In summary, the IWCNS-PSO algorithm introduced in this paper stands out as an efficient system identification and system optimization method, which provides theoretical guidance for the development and debugging of the ultra-flat carrying robot to a certain extent. In the future, the proposed IWCNS-PSO algorithm will be employed in other areas with a view to enhancing the practical effectiveness of the existing work. However, there are still some limitations in this paper, which need to be further discussed. At present, no test has been conducted to verify the simulation results after optimizing the PI parameters, and the simulation results indicate that the control performance of the system are still in need of being further improved, and efforts can be made to modify the control scheme or control strategy, which will be the focus of the follow-up research and the areas to be improved.

## Data availability statement

The original contributions presented in the study are included in the article/supplementary material, further inquiries can be directed to the corresponding author.

## Author contributions

JZ: Formal analysis, Methodology, Software, Writing—original draft, Writing—review & editing. JW: Writing—review & editing, Project administration, Resources, Supervision. ZC: Writing—review & editing, Conceptualization, Data curation. LC: Funding acquisition, Resources, Supervision, Writing—review & editing. MY: Software, Validation, Writing—review & editing. WX: Investigation, Validation, Writing—review & editing.
